# Multiple recurrent aneurysms with angiitis of the central nervous system in a girl: A case report

**DOI:** 10.1097/MD.0000000000032415

**Published:** 2022-12-23

**Authors:** Jiayu Wen, Shengda Ye, Bin Yang, Xi Liu, JinCao Chen

**Affiliations:** a Department of Neurosurgery, Zhongnan Hospial of Wuhan University, Wuhan, China; b Department of Neurology, Zhongnan Hospial of Wuhan University, Wuhan, China.

**Keywords:** angiitis, clipping of intracranial aneurysm, recurrent aneurysm

## Abstract

Primary central nervous system (CNS) vasculitis is a rare immune inflammatory disease confined to the blood vessels of the brain and spinal cord. The total number reported in the world is about 500[1]. Most are negative in cerebral angiography or vascular stenosis and only 1 case of aortic aneurysms reported.

**Patient concerns:**

A 12-year-old female experienced sudden headache and vomiting. Previous findings of vascular stenosis. Diagnosed as a ruptured aneurysm bleeding. The aneurysm recurred a short time after treatment.

**Diagnosis:**

Multiple recurrent aneurysms with angiitis of the central nervous system

**Interventions:**

The patient underwent 2 aneurysm clipping operations, both of which completely clipped the aneurysm.

**Outcomes:**

The patient recovered well after surgery. Three months after discharge, DSA reexamination in our hospital showed that the aneurysm was completely clipped without recurrence.

**Conclusion:**

Subarachnoid hemorrhage after acute cerebral infarction is rare. In addition, the patient had recurrent aneurysms after the first aneurysm clipping, which emphasized the importance of postoperative drug therapy and blood pressure control.

## 1. Introduction

Primary vasculitis of the central nervous system usually affects small and medium blood vessels of CNS. Most showed stenosis and occlusion of intracranial arteries. The total number reported in the world is about 500.^[1]^ After the central blood vessel blocked, hemodynamic changes will be triggered which may lead to cerebral aneurysms and subarachnoid hemorrhage.^[2]^ We report a case of “possible” primary angiitis of the central nervous system (PACNS) in a girl with acute cerebral infarction. The DSA showed that the right middle cerebral artery was stenosed. And a bifurcation segment of the right internal carotid artery aneurysm ruptured with subarachnoid hemorrhage half a year after antiplatelet therapy. The aneurysm reappeared 2 months after the clipping surgery. Finally, after the second craniotomy, the score of NIHSS improved significantly. The purpose of this study is to report the first diagnosed case of recurrent aneurysm as a complication of PACNS.

### 1.1. Case

**Figure 1.**
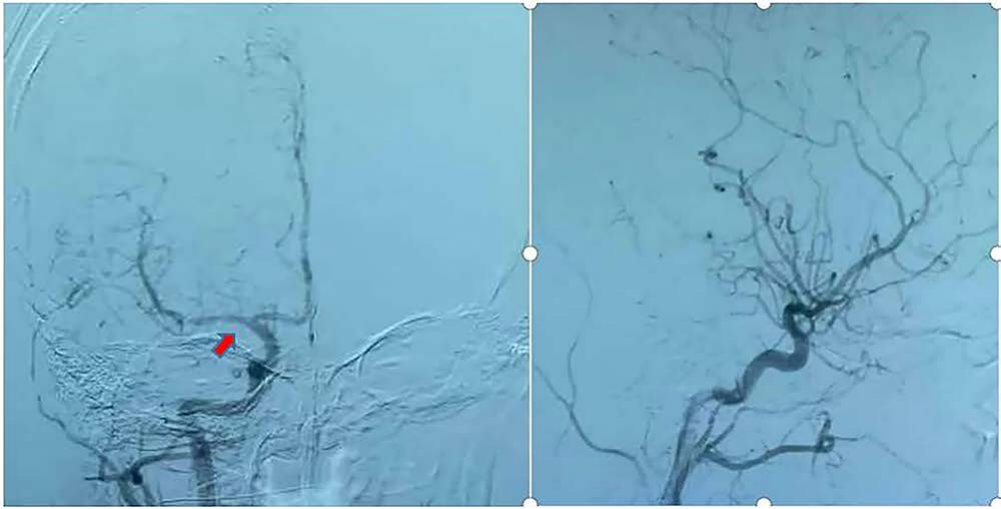
DSA of the patient’s first onset showed stenosis of the right middle cerebral artery without aneurysm formation.

**Figure 2.**
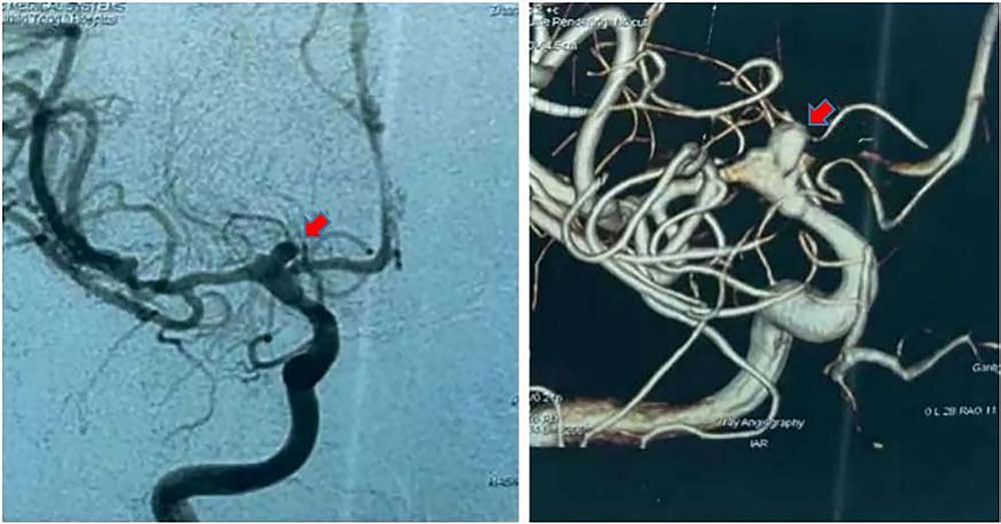
The 3-dimensional reconstructed rotational DSA and DSA of the patient with subarachnoid hemorrhage showed an aneurysm in the bifurcation segment of the internal carotid artery with irregular shape (put arrow on the picture).

**Figure 3.**
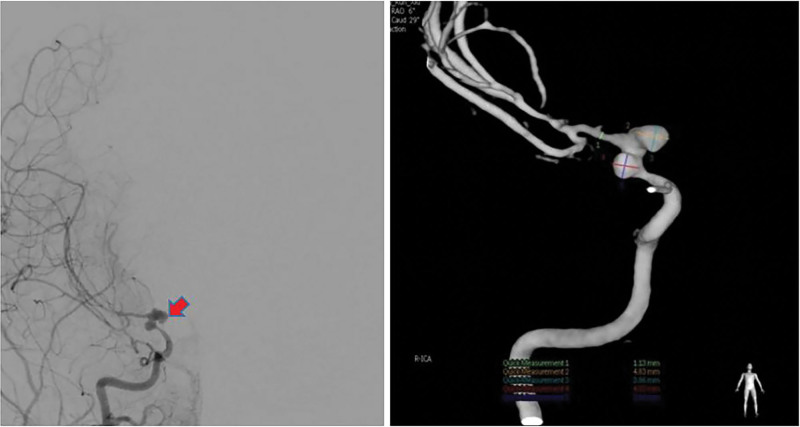
Two months after the first clipping operation, the patient underwent DSA reexamination, which indicated that the aneurysm recurred, and there were 2 aneurysms in the bifurcation of the internal carotid artery.

**Figure 4.**
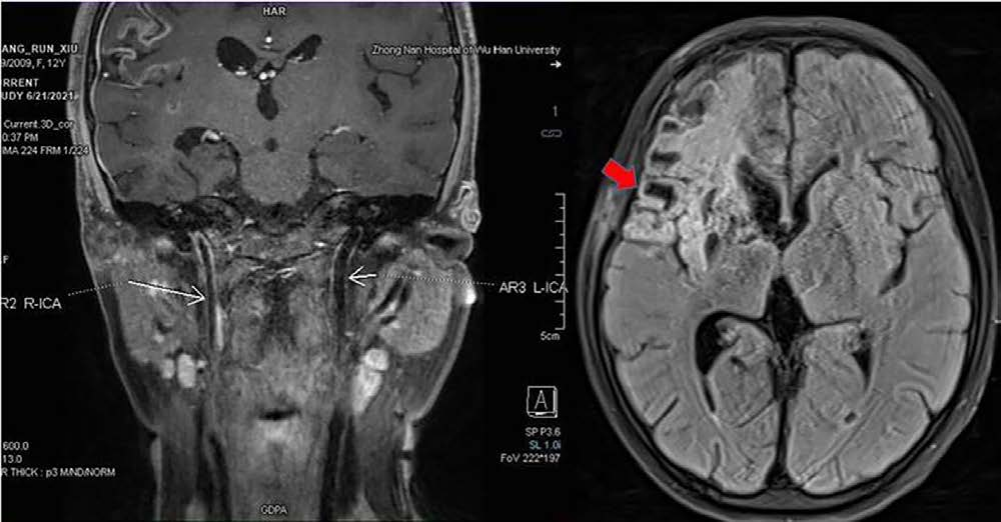
Two months after the first clipping operation, MRI showed stenosis of the right internal carotid artery and white matter damage in right frontal lobe. MRI = magnetic resonance imaging.

The 12-year-old right-handed female patient suddenly suffered from left-sided hemiparesis and unconsciousness. The patient has provided informed consent for publication of the case. And her past history was no special. Magnetic resonance imaging and DSA performed in the local hospital showed acute cerebral infarction with right middle cerebral artery stenosis (Fig. 1). After treatment with indobufen and other drugs, the score of NIHSS was improved. However, 6 months later, the girl developed severe headache with nausea and vomiting. Computed tomography (CT) scan showed subarachnoid hemorrhage in the suprasellar cistern. Cerebral angiography showed occlusion of the right middle cerebral artery and aneurysm of the communicating segment of the right internal carotid artery (Fig. 2). And she received aneurysm clipping. Unfortunately, after 2 months, DSA showed 2 aneurysms in the bifurcation segment of the right internal carotid artery, with sizes of 3.5 mm*2.7 mm and 4.7 mm*3.8 mm (Fig. 3). Therefore, the patient came to our hospital for treatment. Magnetic resonance imaging of the vessel wall confirmed the diagnosis of cerebral white matter damage, vascular stenosis and multiple aneurysms (Fig. 4). But the lumen of the internal carotid artery was too narrow to operate, we finally gave up the endovascular treatment. We also gave the patient positron emission tomography with fluorine-18-fluorodeoxyglucose scan and CT angiography of thoracic and abdominal aorta, no obvious abnormalities were found. We also checked erythrocyte sedimentation rate, thyroid function, direct antiglobulin test, vasculitis related autoantibody, cyclic citrullinated peptide, rheumatoid factor, etc. All the blood inflammatory and immune markers were negative.

One week after stopping the use of oral antiplatelet agents, we followed the original frontotemporal bone flap (pterional approach) to expose the posterior communicating segment and bifurcation of the internal carotid artery. Two original aneurysm clips were seen at the bifurcation, wrapped and tightly adhered to the surrounding tissue. Carefully separated, the original aneurysm clip was taken out and the aneurysm was found. The size of the proximal aneurysm was about 4*6 mm, and the distal about 6*7 mm. Further separate the aneurysm neck, we took multiple aneurysm clips to shape and successfully clipped the aneurysm. Intraoperative fluoroscopy showed that the aneurysm was completely clipped. There were no significant changes in somatosensory and motor evoked potentials in electrophysiological examination. After surgery, the patient was discharged after her condition improved. She was given prednisone, immunosuppression and other support treatment. Three months after discharge, DSA also showed that the aneurysm was well clipped. Cerebrospinal fluid examination, including high throughput gene detection of pathogenic microorganisms, showed no obvious abnormalities and infectious diseases were excluded. And no suspicious gene mutation was found by peripheral blood gene sequencing. The patient was finally diagnosed as “possible” PACNS with intracranial aneurysm due to the lack of histological proof of vasculitis.

## 2. Discussion

Primary vasculitis of the central nervous system can be manifested as focal or diffuse neurological symptoms. At present, little is known about its pathogenesis. PACNS has no peripheral vasculitis and other manifestations of involvement, and most laboratory indicators related to peripheral blood immune inflammation are normal. At present, there is no effective auxiliary examination for diagnosing PACNS. When PACNS is suspected clinically, comprehensive analysis should be carried out by combining the medical history, imaging findings, cerebrospinal fluid analysis results and brain pathology examination results, and similar lesions of PACNS shall be excluded. Serology and cerebrospinal fluid examination are mainly used to exclude secondary central nervous system vasculitis and central nervous system diseases caused by other diseases such as infection or tumor. Calabrese and mallek proposed the diagnostic criteria for PACNS based on clinical experience and evidence from published work.^[3]^ Diagnosis was made if the following 3 criteria were met: a history or clinical findings of acquired neurological deficits of unknown cause after a thorough initial basic evaluation; Cerebral angiography with typical features of vasculitis, or CNS biopsy samples showing vasculitis; And there was no evidence of systemic vasculitis or any other disease that may be secondary to angiographic or pathological features.

PACNS with multiple intracranial aneurysms are extremely rare.^[4]^ In this case, aneurysms occurred successively in the bifurcation of the internal carotid artery shortly after the occlusion of the M1 segment of the middle cerebral artery. It indicated that hemodynamic factors play an important role in the pathogenesis of intracranial aneurysms, which might be caused by the impact of blood flow which lost the original channel. Therefore, as Sun et al emphasized, for the treatment of vasculitis related cerebrovascular disease, we must strictly regulate blood pressure with implement immunotherapy.^[5]^ Aneurysm formation and subarachnoid hemorrhage as related complications of PACNS should be consciously prevented.

When a PACNS patient has severe stenosis of the cerebral arteries and a sudden subarachnoid hemorrhage, we recommend early surgical management of cerebral aneurysm to avoid rebleeding. For the PACNS patients with unruptured cerebral aneurysms, it’s reasonable to consider that surgical treatment should be more active than those with ordinary aneurysms because the elasticity of the vascular walls of PACNS patients have been impaired. The treatment of ruptured cerebral aneurysms should be determined by the indications for intervention or surgery. Intravascular therapy is less intrusive and does not need stopping the dual antiplatelet agents (Indobufene and clopidogrel) that initially used to treat major artery stenosis when both interventional therapy and surgical treatment are available.^[6]^

## 3. Conclusion

We report this medical record for the following reasons. First, the patient’s symptoms are very rare, subarachnoid hemorrhage occurred 8 months after the onset of acute cerebral infarction. Secondly, only 1 previous case report^[2]^ described a large artery aneurysm associated with PACNS and the recurrent aneurysm after aneurysm clipping was the first report, which emphasized the importance of postoperative blood pressure control and drug treatment.AcknowledgmentsWe thank Wenyuan Zhao at Zhongnan Hospital of Wuhan University for his technical support and useful advice.

## Author contributions

All authors have read and agreed to the published version of the manuscript.

**Conceptualization:** Jiayu Wen.

**Data curation:** Jiayu Wen.

**Formal analysis:** Jiayu Wen.

**Funding acquisition:** Shengda Ye.

**Investigation:** Jiayu Wen, Bin Yang.

**Methodology:** Jiayu Wen, Bin Yang.

**Project administration:** Bin Yang.

**Resources:** Bin Yang.

**Software:** Shengda Ye.

**Validation:** Jiayu Wen, Shengda Ye.

**Visualization:** Shengda Ye.

**Writing – original draft:** Jiayu Wen, Shengda Ye.

**Writing – review & editing:** Xi Liu, JinCao Chen.
